# Acute Hypoxic and Refractory Respiratory Failure Induced by an Underlying PFO: An Unusual Case of Platypnea Orthodeoxia and Transient Complication after Transcatheter Closure

**DOI:** 10.1155/2017/4397163

**Published:** 2017-11-28

**Authors:** Carlos Salazar, Romeo A. Majano

**Affiliations:** ^1^Department of Internal Medicine, Weiss Memorial Hospital, 4646 N. Marine Drive, Chicago, IL 60640, USA; ^2^Miami Cardiac and Vascular Institute, South Miami Hospital, Baptist Health, 6200 SW 73 Street, South Miami, FL 33143, USA

## Abstract

Platypnea orthodeoxia (PO) is an infrequent condition of dyspnea with hypoxemia, increased by adopting an upright position and is relieved in decubitus. This condition may occur in patients with hidden intracardiac shunts, usually across a persistent foramen ovale (PFO). The incidence of PFO in general population is quite common, around 27%; however, the concurrent presentation with PO, especially in acute refractory respiratory failure, is extremely rare. PFO closure in this setting is still the treatment of choice with significant improvement or complete resolution of symptoms after closure with an overall periprocedural complication in the first 24 hours of approximately less than 5%. A transient ST-segment elevation in the inferior leads is present in extremely rare occasions and most likely is induced by either an air embolism or a mechanically provoked spasm of coronary arteries. We report a case of an 83-year-old woman in acute hypoxic and refractory respiratory failure in whom PO was identified, most likely induced by a hidden PFO. The patient underwent percutaneous transcatheter closure and developed immediate chest pain, transient hemodynamic instability, and ST-segment elevation in the inferior leads; nevertheless, our patient recovered completely with rapid resolution of respiratory failure with no adverse clinical sequelae.

## 1. Introduction

Platypnea orthodeoxia (PO) is an uncommon presentation of an underlying disorder consisting of hypoxemia and dyspnea upon assuming an upright position and is relieved by decubitus [1]. This condition can be secondary to an intracardiac shunt, a pulmonary vascular shunt, or a ventilation–perfusion mismatch. A right-to-left shunt through a persistent foramen ovale (PFO) may produce a significant desaturation of arterial blood when the patient changes from decubitus to a sitting or standing position [2]. Most PFOs are not expected to cause sufficient right-to-left shunting to elicit hypoxemia due to higher left atrial pressure and greater compliance of the right atrium and ventricle. A spontaneous or induced increase in pulmonary artery pressure (PAP) may produce a sufficient increase in the right atrial pressure to promote a right-to-left shunt ensuing hypoxemia [3]. The incidence of PFO is quite common in general population with an overall incidence of 27% and with a mean diameter of 4.9 mm; however, the concurrent presentation with PO in the context of acute refractory respiratory failure is extremely rare [4, 5]. Transient periprocedural complications in the first 24 hours following PFO closure are uncommon and the pathophysiological mechanisms have not been yet described. We present a case of a patient in acute hypoxic and refractory respiratory failure exhibiting PO due to a right-to-left shunt through a previously undiagnosed PFO. The patient was treated with percutaneous transcatheter PFO closure and presented chest pain, transient hemodynamic instability, and ST-segment elevation after this procedure. The patient recovered completely and there were no adverse clinical sequelae.

## 2. Case Report

An 83-year-old woman with diabetes mellitus type 2, essential hypertension, and hyperlipidemia came to the emergency department with history of 3 days of progressive cough and shortness of breath and 1 day of pressure-like chest pain and anxiety. She did not have a history of smoking. Initial physical examination revealed a patient in severe respiratory distress. Oxygen saturation (O_2_Sat) was 94% with a nonrebreather mask at 80% FIO_2_ and cardiovascular examination revealed a soft 2/6 holosystolic murmur located in the left sternal border, without gallops. Diffused rales were auscultated in both lung fields. The rest of examination and vital signs were unremarkable.

ABG analysis showed acute respiratory alkalosis with moderate hypoxemia and laboratory panels including complete blood count, electrolytes, and troponins were within normal limits. Electrocardiogram (ECG) was normal; chest X-ray and chest computed tomography angiography exhibited diffuse interstitial ground-glass opacities bilaterally suggestive of pulmonary edema and no filling defects ruling out pulmonary embolism. The patient was transferred to the Intensive Care Unit for close monitoring and treated with bilevel positive airway pressure and intravenous diuretics demonstrating a slight clinical improvement; however, hypoxemia persisted and orthodeoxia was identified. O_2_Sat decreased significantly from 91% in decubitus to 86% when adopting a sitting position.

Echocardiography (TTE) showed ejection fraction 50–55% with RVSP 63 and a slightly positive agitated saline contrast test (ASCT) consistent with an interatrial shunting. Transesophageal echocardiography (TEE) was performed showing a positive interatrial communication; nevertheless, images were inconclusive to further evaluate and to determine the precise location of this interatrial defect due to suboptimal echocardiographic images. Cardiac magnetic resonance was not definitive for the presence of an atrial septal defect. Coronary computed tomography angiography was recommended and exhibited mild nonobstructive coronary atherosclerosis and nonspecific pulmonary vascular congestion with no evidence of atrial septal defect or an unroofed coronary sinus.

**Figure 1:**
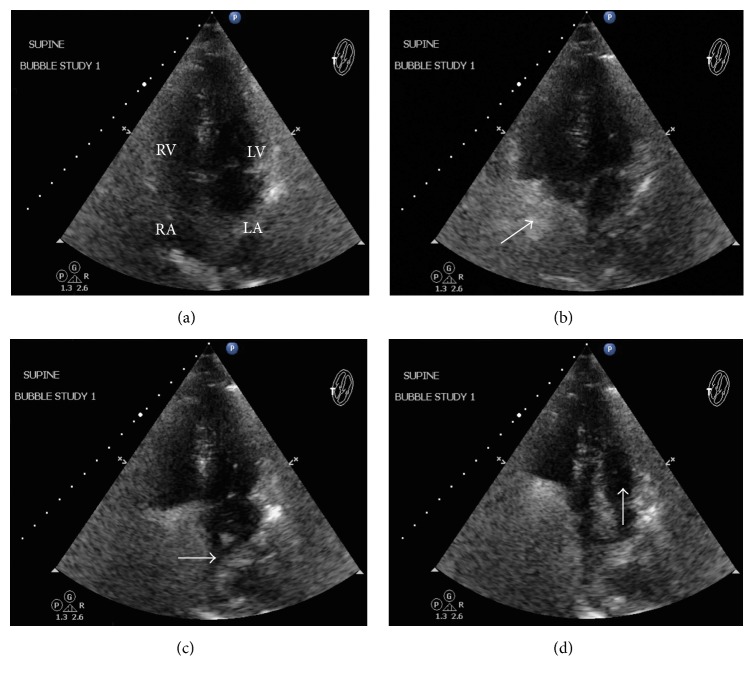
Agitated saline contrast study showing (a) 4 chambers prior contrast infusion. (b) Agitated saline entering the RA. (c) and (d)* Arrows* demonstrating right-to-left shunt into the LA and LV.* RV*: right ventricle;* LV*: left ventricle;* RA*: right atria;* LA*: left atria. Arrows throughout indicate direction of the agitated saline flow.

**Figure 2:**
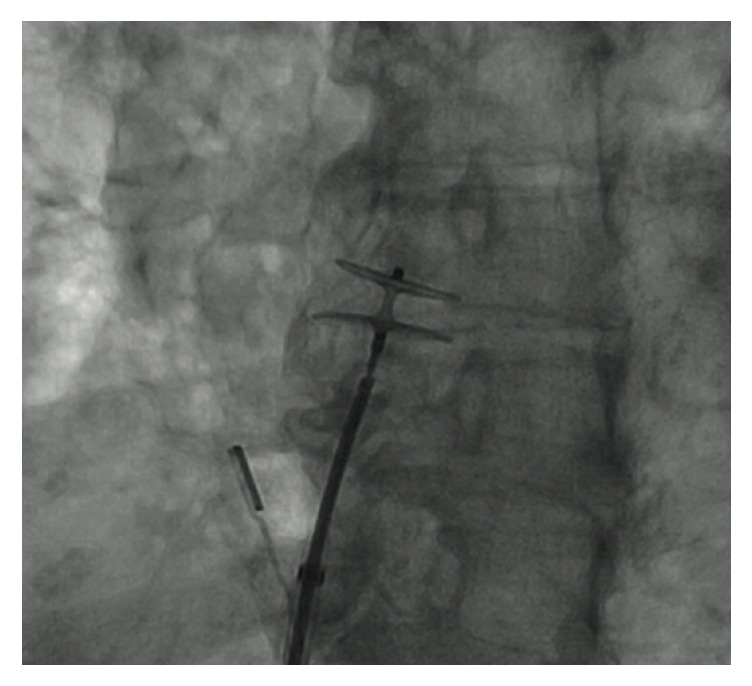
Fluoroscopic positioning and deployment of 18 mm Amplatzer Cribriform Septal Occluder.

Decision was made to clarify the diagnosis with a right heart catheterization and intracardiac echocardiography, which revealed an intact interatrial septum with an evident PFO. An absent “step up” in saturation ruled out a left-to-right shunt. A TTE with ASCT was repeated and performed in reversed Trendelenburg position demonstrating a strongly and obvious right-to-left shunt through a PFO (Figure 1). The defect was emergently closed using an 18 mm Amplatzer Cribriform Septal Occluder (Figure 2). The patient expressed sudden severe chest pain after the procedure. ECG exhibited ST-segment elevation in leads II, III, and aVF, followed by a significant decrease in the mean arterial pressure. Bedsides ultrasonography showed proper device position and no cardiac tamponade was noted. Intra-aortic balloon pump was inserted for hemodynamic support and vasopressors were started. A right coronary artery air embolism was suspected; therefore a coronary angiogram was performed and showed no abnormalities. Left ventricular angiogram and aortogram were within normal parameters.

The patient stabilized with resolution of chest pain and normalization of the ECG changes and became normotensive. A TTE was repeated showing normal left ventricle function, no evidence of pericardial effusion, and no wall motion abnormalities. The patient remained hemodynamically stable, vasopressors were weaned off, and the intra-aortic balloon pump was removed. The patient experienced a positive outcome with complete resolution of respiratory failure with low requirements of oxygen therapy. The patient was discharged to home saturating 98% on room air.

## 3. Discussion

The exact pathophysiological mechanism of PO is not well understood; however, it could be explained by several hypotheses on the basis of positional modifications that favor a shunting phenomenon through an atrial communication. These modifications might increase a right-to-left shunt through a PFO with significant desaturation of arterial blood when the patient stands up or bends over [6]. PFO size tends to be enhanced with increasing age, from a mean of 3.4 mm in the first decade to 5.8 mm in the 10th decade of life [4]. Standing upright might stretch the interatrial communication of the PFO thus allowing more streaming of venous blood from the inferior vena cava through the defect [7]. Another explanation could be a preferential vena caval blood flow directed towards the atrial septum during an upright position, such as in patients with prominent Eustachian valve. In patients with chronic hypoxemia, low mixed venous oxygen tension of blood returning to the pulmonary arteries induces a pulmonary vasoconstriction that increases right atrial pressure and it is believed that it could create a greater gradient of pressures between both atria, supporting a right-to-left shunt through an interatrial communication [8].

PO in the setting of a PFO can be presented with a concomitant increase in PAP; nevertheless, in some cases in which a preferential flow phenomenon has been detected, patients present with normal atrial pressure and PAP but with severe right-to-left shunt [9]. These anatomic abnormalities together with a pulmonary hypertension could deteriorate in an acute, refractory, and dangerous manner a patient's oxygenation status if the diagnosis of a PFO and immediate treatment is not considered. It is imperative to select an initial imaging test for this condition and Muratori et al. in a recent study established that the sensitivity of TTE with ASCT can be improved using provocative measures like valsalva maneuvers [10]. TEE is the gold standard technique for the visualization of atrial septal anatomy, but there might be some technical challenges in eliciting an adequate increase in right atrial pressure during imaging due to varying degree of patient sedation and participation [11]. In our patient, no positional changes were assessed during imaging testing, the strength of the right-to-left shunt through the PFO could have been physiologically reduced during decubitus position and consequently extremely difficult to detect. Transcranial Doppler is another imaging tool with high sensitivity that can be used to screen and quantitate shunt severity; unfortunately, this modality is unable to differentiate cardiac from pulmonary shunts [12].

Percutaneous transcatheter closure is still the treatment of choice for PFO presenting with PO. Guérin et al. demonstrated in 78 patients an immediate increase in saturation and significant decrease in dyspnea following percutaneous occlusion of PFO [13]. A recent study confirmed that 64.8% of patients presenting with PFO and PO had improvement or complete resolution of their dyspnea after transcatheter closure [14]. There have been some studies that have identified that the overall periprocedural complications in the first 24 hours in 276 patients following PFO closure were of 4%. A transient (<3 min) ST-segment elevation, in the inferior leads was present in 1.4%, most likely induced by either an air embolism or a mechanically induced spasm of the coronary arteries [15]. Some authors have reported similar transient events during transseptal catheter procedures such as atrial fibrillation ablation and have hypothesized that following manipulation of the interatrial septum could stimulate left atrial ganglion plexuses causing an imbalance in autonomic innervation, which leads to coronary artery spasm and ST-segment elevation [16]. A recent multicenter, randomized, superiority trial evaluated the procedural complications from PFO closure and demonstrated that atrial fibrillation was only present in 5.9% in comparison to the nonintervention group [17]. Other potential complications of percutaneous device placement can include access difficulties, device embolisation or malposition, pericardial effusion secondary to perforation of the atrium or pulmonary veins, or device thrombus formation [18]. There is no supporting data in literature about sudden chest pain immediately after transcutaneous closing of PFO. A small number of patients have had device removal due to chest pain, presenting late after implantation, of which 18% were associated with nickel allergy present in Amplatzer devices [19]. This would not explain the immediate hemodynamic collapse seen in our patient.

We suspect that the transient hemodynamic instability and ST-segment elevation were probably due to catheter manipulation of the atrial septum or a small air embolism to the right coronary artery, sufficient enough to induce an autonomic imbalance and a possible coronary artery vasospasm. Despite this brief episode of instability, the patient's respiratory failure and chest pain resolved completely and there were no adverse clinical sequelae.

## 4. Conclusion

A PFO as the main cause of acute hypoxic respiratory failure is rare and usually it is not sufficient to cause right-to-left shunting to elicit desaturation. Refractory hypoxemia associated with positional anatomic modifications might favor a shunting phenomenon through an atrial communication. If orthostatic desaturation is detected, it should warrant further investigation. Patients with refractory hypoxemia and suspicious echocardiographic findings should prompt an evaluation with positional changes to facilitate identification of a PFO as a potentially treatable cause of respiratory failure. A noninvasive, safe, and initial diagnosis can be performed by TTE using ASCT moving the patient from a supine to an upright position or by using a tilt-table. Therefore, the shunt can be localized at the atrial level and directly visualized. The transient hemodynamic compromise seen in our patient appeared to be a complication of PFO closure and more studies should be performed to elucidate the exact pathophysiologic mechanism. Physicians should be aware of this transient immediate complication after PFO closure.
